# Impact of nitrogen addition on the chemical properties and bacterial community of subtropical forests in northern Guangxi

**DOI:** 10.3389/fmicb.2024.1418425

**Published:** 2024-08-15

**Authors:** Xingjian Jiang, Zhiyang Ou, Changqiang Tan, Qingfei He, Wei Zheng, Yibo Tan, Feng He, Hao Shen

**Affiliations:** ^1^Guangxi Forestry Research Institute, Nanning, China; ^2^Guangxi Lijiang River Source Forest Ecosystem Research Station, Guilin, China; ^3^Lijiangyuan Forest Ecosystem Observation and Research Station of Guangxi, Guilin, China

**Keywords:** nitrogen deposition, soil bacterial community, soil physicochemical properties, subtropical areas, Mao’er Mountain

## Abstract

**Introduction:**

In recent years, nitrogen deposition has constantly continued to rise globally. However, the impact of nitrogen deposition on the soil physicochemical properties and microbial community structure in northern Guangxi is still unclear.

**Methods:**

Along these lines, in this work, to investigate the impact of atmospheric nitrogen deposition on soil nutrient status and bacterial community in subtropical regions, four different nitrogen treatments (CK: 0 gN m^–2^ a^–1^, II: 50 gN m^–2^ a^–1^, III: 100 gN m^–2^ a^–1^, IV: 150 gNm^– 2^ a^–1^) were established. The focus was on analyzing the soil physical and chemical properties, as well as bacterial community characteristics across varying nitrogen application levels.

**Results and discussion:**

From the acquired results, it was demonstrated that nitrogen application led to a significant decrease in soil pH. Compared with CK, the pH of treatment IV decreased by 4.23%, which corresponded to an increase in soil organic carbon and total nitrogen. Moreover, compared with CK, the soil organic carbon of treatment IV increased by 9.28%, and the total nitrogen of treatment IV increased by 19.69%. However, no significant impact on the available nitrogen and phosphorus was detected. The bacterial diversity index first increased and then decreased with the increase of the nitrogen application level. The dominant phylum in the soil was *Acidobacteria* (34.63–40.67%), *Proteobacteria*, and *Chloroflexi*. Interestingly, the abundance of *Acidobacteria* notably increased with higher nitrogen application levels, particularly evident in the IV treatment group where it surpassed the control group. Considering that nitrogen addition first changes soil nutrients and then lowers soil pH, the abundance of certain oligotrophic bacteria like *Acidobacteria* can be caused, which showed a first decreasing and then increasing trend. On the contrary, eutrophic bacteria, such as *Actinobacteria* and *Proteobacteria*, displayed a decline. From the redundancy analysis, it was highlighted that total nitrogen and pH were the primary driving forces affecting the bacterial community composition.

## 1 Introduction

Nitrogen is essential for plant growth as it is a key element in various plant components including chlorophyll, proteins, and enzymes. It plays a crucial role in regulating plant metabolism and activities (Xu et al., 2019). A large amount of nitrogen is needed to maintain plant growth. However, atmospheric nitrogen deposition has significantly increased from industrialization, and nitrogen deposition will alarmingly continue to increase in the coming decades. The increase of nitrogen usually leads to enhanced productivity of plant communities by increasing the available nutrients. Nonetheless, excess nitrogen may lead to limitations of other nutrient elements, such as phosphorus, thus reducing the richness and diversity of plant species (Gao et al., 2022). There are also concrete pieces of evidence indicating that excessive nitrogen input has caused harm to the ecological environment, such as degradation of freshwater resources (Baron et al., 2013), soil acidification, and increased loss of soil organic nitrogen (Brookshire et al., 2007), The crop growth has been harmed (Cheng et al., 2020), the soil greenhouse gas release rate has been affected (He et al., 2021), plant mortality has been increased, and microbial community structure has been changed (Zak et al., 2019; Moore et al., 2021). Thus, it is apparent that it threatens the species diversity and balance of forest ecosystems. The meta-analysis of ^15^N labeling experiments in the whole forest ecosystem showed that most of the deposited nitrogen accumulated in the soil ecosystem (Templer et al., 2012). The subtropical region in China is considered one of the hot zones of N deposition in the world (Du et al., 2019; Du, 2020). Notably, subtropical old-growth forests are irreplaceable and have greater resilience to disturbance than other forests (Watson et al., 2018). Forest ecosystems are the main recipients of N deposition, and the impact of N deposition on the structure and function of forest ecosystems in the context of global change has been extensively investigated. Consequently, the impact of N addition on the structure and function of the subtropical evergreen broad-leaved forests has been explored (Tian et al., 2022). More specifically, the effects of N deposition on biodiversity (Lu et al., 2010; Tian et al., 2019), soil biochemistry (Shi et al., 2016; Lu et al., 2021), and plant nutrient stoichiometry and functional traits (Lu et al., 2018; Zhu et al., 2019; Tang et al., 2021) has been examined. However, limited experiments have been conducted on the influence of nitrogen deposition in subtropical old-growth forests in Guangxi. Therefore, it is of vital importance to deeply understand the response of soil ecosystems to global change by understanding the impact of nitrogen deposition on the change of soil nutrients and the diversity of soil bacteria.

With the aggravation of nitrogen deposition, the relationship between soil nutrient cycling and nutrients has also been greatly affected. Nevertheless, the connection between nitrogen deposition and soil nutrients remains elusive. Some works in the literature have found that atmospheric nitrogen deposition can reduce soil pH, resulting in soil acidification (Wang et al., 2023b). The change in the nitrogen content can also affect soil nitrogen mineralization and nitrification, and the addition of nitrogen can lead to an increase in the soil available nitrogen (Liu et al., 2017). Other works have reported that nitrogen deposition did not increase the soil available nitrogen content (Hu et al., 2010; D’Orangeville et al., 2014). The impact of nitrogen addition on organic carbon also varied with different ecological environments. Most forest ecosystems showed an increasing trend (Yang et al., 2023). It has been also demonstrated that nitrogen addition had no significant impact on organic carbon (Hu et al., 2010). Although the application of nitrogen significantly increased the content of soil soluble organic carbon (Wang et al., 2008; Xu et al., 2023), it was found that nitrogen fertilizer reduced the content of soluble organic carbon in the northern region (Zhang et al., 2019). It was also found that low nitrogen had a promoting impact on soluble carbon. The addition of high nitrogen inhibited the content of soluble carbon (Shi et al., 2019). Regarding the relationship between nitrogen addition and phosphorus content, some works in the literature have concluded that nitrogen addition reduces the soil total phosphorus content but has no significant impact on the available phosphorus (Tian et al., 2020). In another work, it was reported that nitrogen does not significantly affect both total phosphorus and available phosphorus (Li et al., 2013). The relationship between the nitrogen addition and soil physical and chemical properties is affected by different regions and different climate conditions. Therefore, further research is required to shed light on the underlying origins of this relationship.

Soil microorganisms participate in almost all material transformation processes in the soil and connect the material cycles of soil, biosphere, atmosphere, hydrosphere, and lithosphere. They can release nutrients from minerals and organic matter, fix nitrogen, and maintain the stability of soil aggregates (Adingo et al., 2021). They serve as crucial mediators and regulators in the cycling of soil metal and nonmetal elements, exerting profound influence on the overall health and functionality of soil ecosystems (He et al., 2024). In forest ecosystems, soil bacteria account for the vast majority of soil microbial communities. Changes in bacterial community structure and composition can quickly reflect changes in soil conditions (Li et al., 2004). Soil bacterial activity and community composition directly regulate the soil carbon cycle and turnaround process (Zechmeister-Boltenstern et al., 2011). Many works have examined the impact of nitrogen deposition on soil bacterial communities. Previous meta-analyses have shown that nitrogen deposition has a negative impact on soil microorganisms in terrestrial ecosystems including forest ecosystems, reducing microbial diversity (Zhang et al., 2018; Yang et al., 2020, 2022). Other works have found that fertilization can increase the diversity and richness of soil bacteria (Arunrat et al., 2023). It has been also proven that nitrogen addition has a negligible impact on soil microbial communities in forest ecosystems but can affect sensitive bacterial groups (He et al., 2023). There are also a few works that have found that soil microbial communities and structures in grassland and desert ecosystems are not affected by nitrogen content (Fierer et al., 2012; Sha et al., 2021). This result points out that nitrogen addition affects soil microorganisms that are influenced by ecosystem heterogeneity. Now, most nitrogen addition experiments are conducted in temperate and boreal forests (Tian et al., 2017). However, the relationship between the underground bacterial communities and nitrogen addition in natural forests in subtropical areas with frequent human activities, large amounts of nitrogen deposition, and rich species has not been systematically explored. However, in humid areas, acidic soils in subtropical forests are more sensitive to nitrogen deposition than in temperate forests (Lu et al., 2014). Mao’er Mountain is located at the source of the Li River and on the Xianggui Corridor. Every summer, warm and humid air from the Beibu Gulf flows northward, blocked by peaks represented by Mao’er Mountain and gradually climbs, resulting in a decrease in temperature, and forming terrain rain. Warm and humid air currents sometimes meet with cold air moving southward along the Xianggui Corridor, forming frontal rain. The special terrain that makes Mao’er Mountain nearby rainfall is particularly abundant, making it one of the rainfall centers in Guangxi. This effect could induce the deposition of nitrogen in the atmosphere in this area, necessitating the conduction of nitrogen addition experiments. The current research in this area has focused on the differences in plant growth characteristics and physical and chemical properties at different altitudes or different tree species. However, the impact of nitrogen addition on soil physical and chemical properties and bacterial community structure remains elusive (Song et al., 2016; Tan et al., 2019, 2023; Dong et al., 2020). A deep understanding of the changes in soil nutrient status and bacterial community structure of natural forests in this region under different nitrogen treatments will help the sustainability of the regional ecosystem. A solid scientific basis for forest management measures in response to future nitrogen deposition will be also provided.

Under this direction, this work aims to test the impact of different N addition rates on the soil chemical properties and bacterial communities. Our analysis is based on three objectives: (1) examine the impact of N addition on soil chemical properties; (2) explore the impact of N addition on bacterial communities and (3) study the potential mechanisms associated with changes in soil properties and bacterial communities. It was hypothesized that: (1) N addition increases soil nutrient content and reduces pH; (2) N addition reduces bacterial diversity and alters bacterial community composition and (3) pH is the main driving factor affecting the structure of bacterial communities.

## 2 Research area and methods

### 2.1 Overview of the study area and experimental design

The research area is located in Mao’er Mountain National Nature Reserve in Guilin, Guangxi (110°19′∼ 110°31′E, 25°44′∼ 25°58′N), the highest peak in South China, the main peak of the Nanling Mountains (Tan et al., 2023). It is also the birthplace of Lijiang River, Zijiang River and Xunjiang River (Tan et al., 2019). The region is a humid mountain monsoon climate zone in the middle subtropics, with high temperature and rain in summer, mild and little rain in winter, annual precipitation above 3,000 mm, and the main soil types are red and yellow soil series.

The plots were set in a subtropical evergreen broad-leaved forest at an altitude of 1,100 m in Mao’er Mountain. Four nitrogen application levels were set up in the plots, the nitrogen application rate is set according to the commonly used nitrogen application rates in subtropical regions with similar geographical locations (Qiu et al., 2023), namely, 0 gN⋅m^–2^⋅a^–1^, 50 gN⋅m^–2^⋅a^–1^, 100 g N⋅m^–2^⋅a^–1^, 150 gN⋅m^–2^⋅a^–1^, and five repeated quadrats were set up for each treatment. The quadrat size was 5 m × 5 m. The spacing between each quadrat was 5 m to avoid cross effects between the various treatments. Nitrogen treatment was started in July 2017, and nitrogen was applied once every 2 months. Ammonium nitrate was dissolved in water and evenly sprayed into the sample plot. An equal amount of clean water was applied to the control treatment. The main tree species in the sample plot were *Lithocarpus hancei (Bentham) Rehd*, *Alniphyllum fortunei (Hemsl.) Makino*, *Daphniphyllum macropodum*, *Eurya brevistyla Kobuski* and *Rhododendron simsii Planch*, etc. Main shrub were *Symplocos anomala*, *Ilex chinensis Sims*, *Rhododendron simsii Planch*, *Eurya brevistyla Kobuski* and *Rhododendron cavaleriei Levl*, etc. Major herbs were *Woodwardia japonica*, *Fordiophyton fordii*, *Hicriopteris chinensis*, *Tripterospermum chinense* and *Smilax china L*, etc.

### 2.2 Soil sample collection

In July 2019, slopes with roughly the same slope direction were selected, and soil samples were collected using a soil auger at a sampling depth of 0–20 cm. The soil was taken from 5 random points in each quadrat and mixed evenly. A total of 4 (different nitrogen application treatments) × 5 (repeated) = 20 soil samples were collected. Part of the collected samples were put into ziplock bags for physical and chemical property determination. Moreover, part of them was passed through a 2 mm sieve and stored in liquid nitrogen for cryogenic storage for subsequent DNA extraction.

### 2.3 Testing method

The pH value was measured by potentiometric method (soil-to-water ratio is 1:2.5); the soil organic carbon (SOC) was measured by potassium dichromate hydration heating-ferrous sulfate titration method; the soil organic matter (SOM) equals 1.724 multiply SOC; the total nitrogen (TN) was measured by H_2_SO_4_ digestion-Kjeldahl method. The soil ammonium nitrogen was measured using 2 mol⋅L^–1^ KCl extraction-indophenol blue colorimetric method; The nitrate nitrogen was measured using dual-wavelength ultraviolet spectrophotometry, and soil available phosphorus (AP) was detected using the molybdenum-antimony colorimetric method. The soil total phosphorus (TP) was measured using sulfuric acid-perchloric acid digestion and leaching and molybdenum-antimony colorimetric methods (Bao Shidan, 2000). The soil soluble carbon (DOC) was extracted with 0.5 mol L^–1^ K_2_SO_4_ (soil-water ratio 1: 4). After the extract was filtered by 0.45 μm membrane, the total organic carbon in the filtrate was determined by TOC-L CPH total organic carbon analyser (Shimadzu, Japan, the detection limit was 2 μ mol L^–1^) (Haynes, 2000).

MoBio’s Power Soil TM DNA Isolation Kit (MoBio, USA) was used for DNA extraction. The integrity of DNA was determined by 1% agarose gel electrophoresis, and the concentration and purity of DNA were determined by Mini Drop. Ling En biological company IlluminaPE250 high-throughput sequencing platform was used for sequencing. The sequencing region was the 16S V3V4 region of standard bacteria, the upstream primer was 338F, and the upstream primer sequence was 5′-ACTCCTACGGGACGCAGCA-3′, downstream primer was 5′-CGGACTACHVGGGTWTCTAAT-3′. The DNA amplification conditions were pre-denatured at 98°C for 1 min, denatured at 98°C for 10 min, annealed at 50°C for 30 cycles at 72°C, and extended for 5 min at 72°C. The sequencing library was prepared by TruSeq Nano DNA LT Library Prep Kit of Illumina company. The final fragments of the library were selected and purified by 2% agarose gel electrophoresis. The Illumina Miseq platform was used for sequencing. Microbiological data can be obtained from the NCBI website (No: PRJNA1062461).

### 2.4 Data processing

The WPS table was used for data sorting and calculation; Minitab19 was used for difference analysis, The significant difference was explored by Tukey’s Honestly Significant Difference (HSD) test; prism8.0 was used to draw correlation analysis diagrams; Pearson rank was used for the correlation analysis; Canoco5.0 was used to draw redundancy analysis diagrams; the ggven packages in the R language were used to draw Venn map; the vegan packages in the R language were used to draw the NMDS map.

## 3 Results and analysis

### 3.1 Impact of different nitrogen application levels on soil physical and chemical properties

The soil physical and chemical properties among the different nitrogen application treatments are listed in Table 1. The soil pH content under the IV nitrogen application level was significantly lower than that of other nitrogen application levels and control (*P* < 0.05). Compared with CK, the pH of treatment IV decreased by 4.23%. The nitrogen application level was II class, the pH value was higher than that of the control, and decreased with the increase of the nitrogen application rate. The content of soil organic carbon and organic matter under the IV nitrogen application level was significantly higher than that of the control (*P* < 0.05). Compared with CK, the SOC of treatment IV increased by 9.28%. With the increase in the nitrogen application rate, the content of the soil organic carbon and organic matter increased. The soil soluble organic carbon at the II level was significantly higher than that at the IV level (*P* < 0.05). With the increase in the nitrogen application rate, the soluble organic carbon showed a downward trend. The total soil nitrogen under level IV nitrogen fertilization level was significantly higher than other nitrogen fertilization treatments (*P* < 0.05). In addition, the TN of treatment IV increased by 19.69% and nitrogen showed an increasing trend with the increase of the nitrogen fertilization amount. No significant difference in soil ammonia nitrogen among the different nitrogen application treatments was detected. However, it increased with the increase of the nitrogen application rate. Although the soil nitrate nitrogen had no significant difference among the different nitrogen application rates, it first increased and then decreased with the increase of the nitrogen application rate. There were also significant differences in soil total phosphorus between the different nitrogen fertilization treatments. The total phosphorus content in the IV nitrogen fertilization treatment was significantly lower than the control (*P* < 0.05). Significant differences in the soil total phosphorus among the different nitrogen application treatments were recorded. In particular, the total phosphorus content under the grade IV nitrogen application was significantly lower than that under control (*P* < 0.05). Furthermore, the total phosphorus content under the grade III nitrogen application level was significantly higher than that under grade IV nitrogen application level (*P* < 0.05). Total phosphorus showed a trend of first increasing and then decreasing with the increase of the nitrogen application rate. No significant difference in soil available phosphorus among the different nitrogen application treatments was detected.

**TABLE 1:** Soil physical and chemical properties under different nitrogen addition levels.

Nitrogen addition levels	pH	SOC (g/kg)	SOM (g/kg)	DOC (mg/kg)	TN (g/kg)	NH_4_^+^-N (mg/kg)	NO_3_^–^-N (mg/kg)	TP (g/kg)	AP (mg/kg)
CK	4.26 ± 0.08A	38.57 ± 1.60B	66.50 ± 2.76B	23.09 ± 3.86AB	1.93 ± 0.13B	18.36 ± 1.36A	2.37 ± 0.32A	0.54 ± 0.03A	1.25 ± 0.28A
II	4.29 ± 0.04A	40.27 ± 1.84AB	69.43 ± 3.17AB	27.81 ± 5.35A	1.89 ± 0.21B	14.03 ± 2.89A	2.18 ± 0.37A	0.45 ± 0.05BC	1.18 ± 0.30A
III	4.21 ± 0.05A	41.36 ± 2.18AB	71.30 ± 3.77AB	21.41 ± 3.34AB	1.94 ± 0.20B	16.60 ± 3.43A	3.09 ± 0.30A	0.50 ± 0.02AB	1.40 ± 0.44A
IV	4.08 ± 0.08B	42.15 ± 1.78A	72.67 ± 3.08A	20.06 ± 2.57B	2.31 ± 0.22A	18.34 ± 3.69A	2.96 ± 0.93A	0.42 ± 0.03C	0.93 ± 0.19A

Different capital case letters in the same column indicate significant differences (*P* < 0.05). Data in table are mean ± standard deviation.

### 3.2 Soil bacterial diversity and community composition under different nitrogen application levels

**FIGURE 1:**
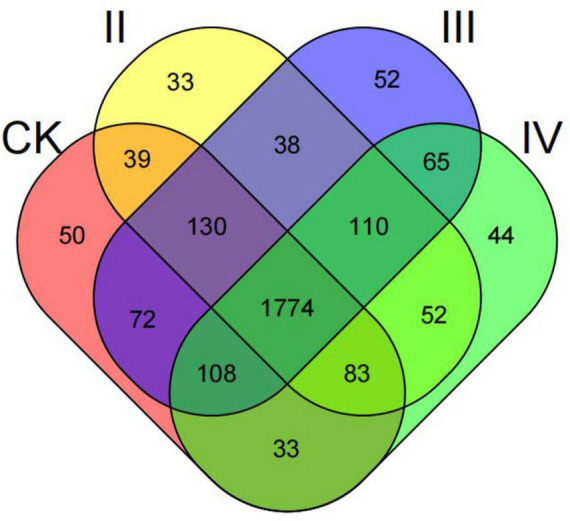
Venn diagram showing the unique and shared OTUs at different nitrogen treatments in bacterial communities.

The total number of OUT was 2,683, and the common OUT was 1,774 (Figure 1). Among them, the unique OUT numbers of nitrogen application levels of CK, II, III, and IV were 50, 33, 52, and 44, respectively, accounting for 1.86, 1.23, 1.94, and 1.64% of the total OUT, respectively. By performing a pairwise comparison, it was found that the total number of OUT at CK and III levels was up to 2,084. Under different nitrogen application levels, the number of single OUT was CK2289, II2259, III2349, and IV2269, respectively. With the increase in the nitrogen application rate, the number of OUT first increased and then decreased. The number of OUT after nitrogen application under II and IV levels was lower than that of the control, and the number of OUT under III nitrogen treatment was 2.62% higher than that of the control.

**FIGURE 2:**
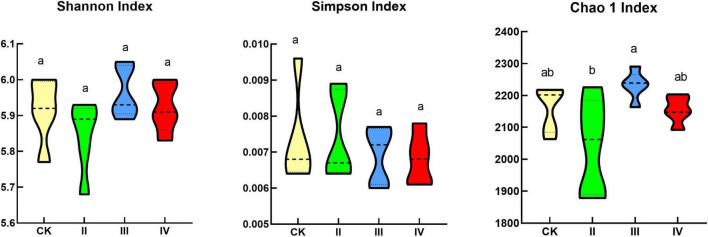
Bacterial diversity index. Different lowercase letters indicate significant differences under different nitrogen application treatments (*P* < 0.05).

There was no significant difference between the Shannon index and Simpson index among the different nitrogen treatments (Figure 2). More specifically, the Shannon index and Simpson index first increased and then decreased with the increase of the nitrogen application. The Shannon index and Simpson index were lower than the control at grade II and IV nitrogen application levels; under III level of nitrogen application, the Shannon index and Simpson index were higher than those of the control. The Chao1 index at the III nitrogen application level was significantly higher than that at the II nitrogen application level; with the increase in the nitrogen application rate, the Chao1 index first increased and then decreased; The Chao1 index at the II and IV nitrogen application levels was lower than the control; under the III nitrogen application level, the Chao1 index was higher than the control.

**FIGURE 3:**
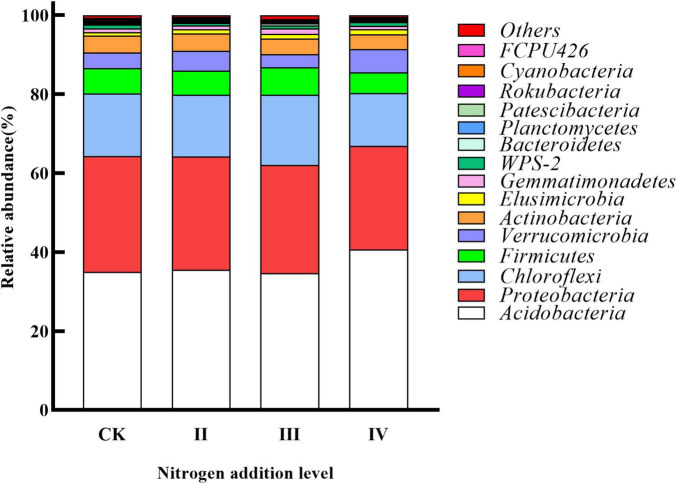
Relative abundance of soil bacterial phylum levels under different nitrogen addition levels.

*Acidobacteria*, *Proteobacteria*, *Chloroflexi*, *Firmicutes*, and *Verrucomicrobia* were the main dominant bacteria (Figure 3). The response of the different bacterial groups to nitrogen addition was different. The abundance of *Acidobacteria* increased with the increase of the nitrogen application, while the abundance of *Proteobacteria* and *Actinobacteria* decreased with the increase of the nitrogen application. The relative abundance of *Chloroflexi* and *Firmicutes* first increased and then decreased; the relative abundance of *Verrucomicrobia* first decreased and then increased. Compared with the control without nitrogen application, the abundance of *Acidobacteria* at the IV level was higher than that of the control.

### 3.3 Impact of nitrogen addition on soil bacterial community structure

**FIGURE 4:**
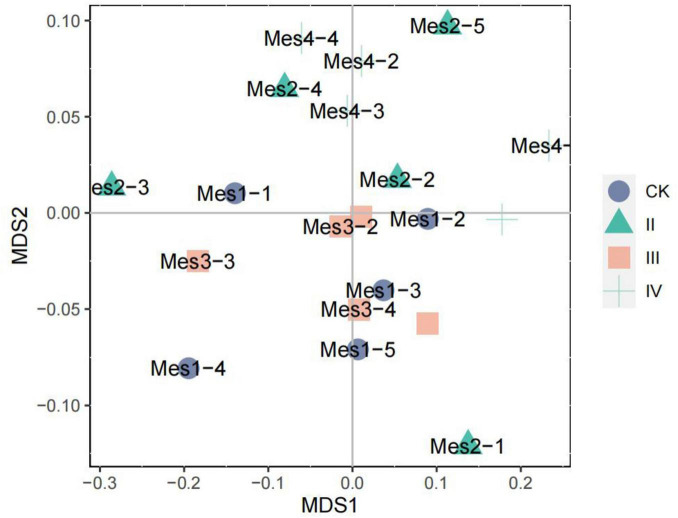
NMDS analysis of soil bacterial community.

As can be seen from Figure 4, the high nitrogen addition has a certain effect on the soil bacterial community structure. At the level of IV nitrogen application, the soil bacterial community structure is similar. However, different nitrogen treatments do not cause great change to the community structure, and the community structure does not appear obvious separation. Under the level of CK and III nitrogen application, the soil bacterial community was mainly distributed on the lower right side of NMDS. The soil bacterial community structure without nitrogen fertilizer (CK) was similar to that under III nitrogen application, indicating that there may be the same species. Under the level of II nitrogen application, the structure of the bacterial community was scattered.

### 3.4 Correlation analysis between the soil bacterial α diversity and the physicochemical properties under different nitrogen application levels

**FIGURE 5:**
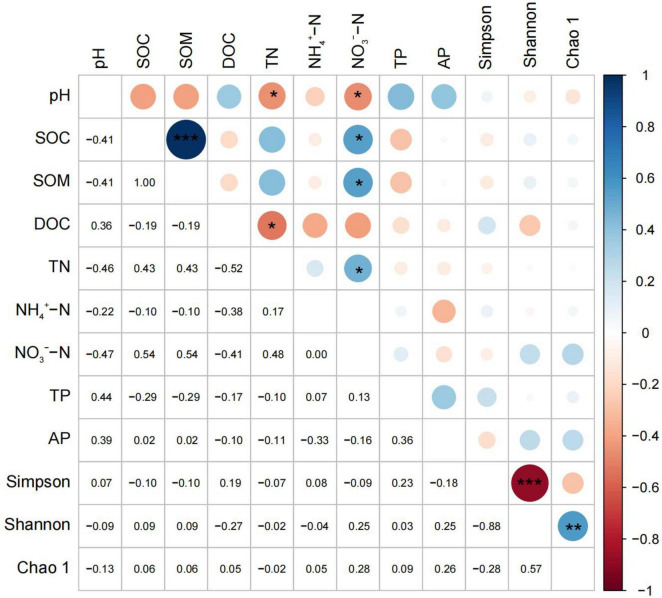
Correlation analysis between soil physicochemical properties and bacterial diversity. Significant correlation are indicated by “*”, “**” or “***” according to Pearson correlation test (*P < 0.05, **P < 0.01, and ***P < 0.001).

The correlation analysis showed that soil pH was negatively correlated with the total nitrogen and nitrate nitrogen. The soil organic carbon was positively correlated with the soil organic matter and nitrate nitrogen and the soil organic matter was positively correlated with the nitrate nitrogen. Additionally, the soluble organic carbon was negatively correlated with the total nitrogen, and the soil nitrogen was positively correlated with the nitrate nitrogen (Figure 5). No correlation between the ammonium nitrogen and organic carbon and organic matter, as well as between the soluble organic carbon and phosphorus was detected. No correlation between the nitrate nitrogen and the available phosphorus was also found. There was a significant positive correlation between the Simpson index and the Shannon index, and a significant negative correlation between the Shannon index and the Chao1 index. There was also no significant difference between the soil diversity and soil physical and chemical properties.

### 3.5 Redundancy analysis between the soil physicochemical properties and bacterial community under different nitrogen application levels

**FIGURE 6:**
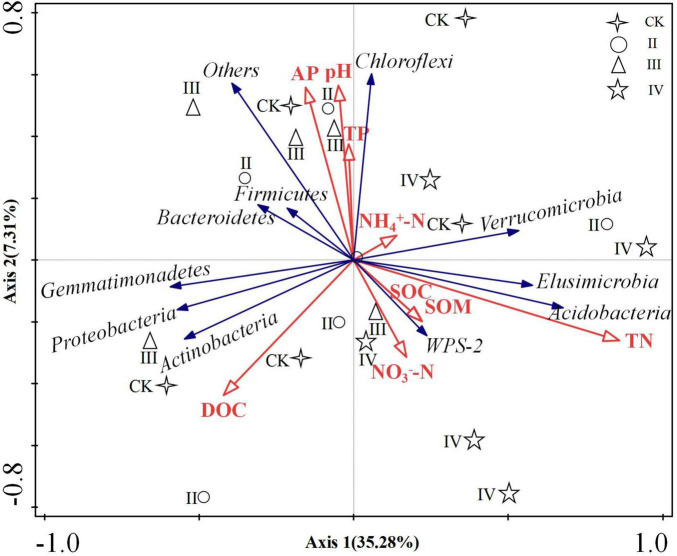
Analysis of soil physicochemical properties and bacterial community redundancy.

The soil physical and chemical properties and bacterial diversity index were used as explanatory factors, and the dominant flora at the bacterial phylum level was used as response variables for redundancy analysis (Figure 6). The degree of explanation of the first axis was 35.28%, and that of the second axis was 7.31%. The common explanation of the two axes is 39.96%. The soil total nitrogen is considered the key factor driving soil bacterial community structure, and its explanation degree was 26.4%, *P* = 0.004. The second driving factor is pH, and its explanation degree was 6.5%. The total nitrogen was positively correlated with *Acidobacteria*, *Elusimicrobia*, *Verrucomicrobia*, and *WPS-2*, and negatively correlated with *Actinobacteria*, *Firmicutes*, and *Proteobacteria*.

## 4 Discussion

### 4.1 Impact of nitrogen addition on the soil physical and chemical properties

In our work, nitrogen addition has a significant impact on some soil nutrients. When the nitrogen application level was level IV, the soil pH was significantly reduced, and was negatively correlated with total nitrogen. The results of this work are also in good agreement with the majority of the reported outcomes in the literature (Hu et al., 2010; Li et al., 2018; Tian et al., 2018; Wang et al., 2023a). This is mainly because nitrogen addition decreased the amount of exchangeable cations in soil, resulting in soil acidification. Our correlation study also showed the existence of a significant negative correlation between the pH and nitrate nitrogen (Figure 5, *P* < 0.05). This effect indicates that nitrogen addition led to the increase of both the nitrate nitrogen content and nitrate ions in the soil, resulting in soil acidification.

It was also proven that nitrogen addition increased soil organic carbon and organic matter content. When the nitrogen application level was level IV, the organic matter and organic carbon content were significantly higher than the control. This result is consistent with the previously reported works in the literature suggesting that nitrogen application in forest ecosystems can promote the accumulation of soil organic matter (Yang et al., 2023). When the nitrogen application rate was level IV, the soil nitrogen content was also significantly higher than the control and other nitrogen application levels. The reason for this phenomenon may be the increase in root productivity and the acceleration of litter decomposition after nitrogen enrichment. It is well-known that organic matter components enter the soil (Zhang et al., 2023). In addition, the increase in nitrogen content will improve the nutritional status of litter and increase the activity of carbon and nitrogen hydrolases in the soil. This, accumulation of soil nitrogen and organic matter will be promoted (Yang et al., 2022; Xu et al., 2023).

Our work found that soluble organic carbon was higher than the control under the level II nitrogen fertilization level. As the amount of nitrogen application increased, DOC showed a downward trend. Under the level IV nitrogen fertilization level, DOC was significantly lower than the level II nitrogen fertilization level, indicating that low nitrogen increases DOC content. In striking contrast, medium and high nitrogen suppresses DOC concentration. This research result is consistent with the outcomes of Yuan (Yuan et al., 2022) who studied the impact of nitrogen addition on the soluble organic carbon content of Taiwanese pine soil in subtropical areas. From the nitrogen addition in Northeast China, it was found that low nitrogen promoted soluble organic carbon while high nitrogen inhibited it. These results of the DOC content are consistent with (Shi et al., 2019). The 7-year long-term nitrogen addition test also showed that the DOC content was significantly reduced (Lu et al., 2013). This effect may be because nitrogen application can promote the degradation of refractory organic matter. As a result, a large amount of DOC will be produced after low nitrogen addition. In high-nitrogen soils, the increase in microbial activity induced by nitrogen addition leads to faster DOC absorption (Xu et al., 2023). N addition, soil acidification after high nitrogen addition inhibits enzyme activity and thus reduces DOC (Zak et al., 2011).

Our work concluded that different nitrogen application levels had no significant impact on soil available nitrogen. In particular, the content of ammonium nitrogen after nitrogen addition was lower than that of the control group. This result is inconsistent with the previously reported outcomes indicating that nitrogen addition significantly increased the soil available nitrogen (Zeng et al., 2016; Wang et al., 2018c). It can be speculated that this effect may be due to the rapid uptake and utilization of available nitrogen by plants after nitrogen enrichment, especially for ammonium nitrogen. Relevant works have also shown that plants will absorb easily available nitrogen in the soil after nitrogen addition, resulting in a significant increase in nitrogen content in leaves (Tian et al., 2018).

It was also found that when the nitrogen application level was level IV, the soil total phosphorus was significantly lower than the control. In parallel, the available phosphorus did not show significant differences with changes in nitrogen application amount. Previous long-term nitrogen addition experiments also confirmed this result. The nitrogen addition intensified the phosphorus limitation that occurs in a variety of terrestrial ecosystems including tropical forests, possibly because nitrogen enrichment stimulates plant uptake of phosphorus (Wang et al., 2023c). Another reason may be that the activity of phosphorus-solubilizing bacteria in the soil decreases after nitrogen is added (Wang et al., 2023a). When the pH decreases, the activity of alkaline phosphatase will also be inhibited, resulting in a decrease in the phosphorus content. When the nitrogen application rate was 150 gN⋅m^–2^⋅a^–1^, some soil chemical properties underwent significant changes. This effect points out that the current nitrogen application rate in the Central Asian hot region has reached the threshold for changing physical and chemical properties. When the local nitrogen content exceeds the threshold, the nitrogen input should be controlled to reduce the harm to the forest.

### 4.2 Impact of nitrogen addition on soil bacterial diversity and composition

Our work found that with the application of nitrogen fertilizer levels, the total OUT number showed a trend of first increasing and then decreasing, indicating that an appropriate amount of nitrogen input could improve the kind of soil bacterial. However, excessive nitrogen input can inhibit the kind of soil bacteria. The reason for this effect could be ascribed to the low nitrogen input that can accelerate the decomposition of litter and promote the process of nutrient cycling by providing nutrition for the growth and development of microorganisms. Hence, the growth of microorganisms is promoted (Zhang et al., 2023). Excessive nitrogen content will lead to soil acidification, which is not conducive to the growth and survival of microorganisms (Yang et al., 2020). After adding nitrogen, the growing soil environment of microorganisms will become more harsh. Taking into account that most bacteria have poor tolerance to soil pH, a decrease in the number and diversity of bacteria will be induced (Wang et al., 2018d).

Simpson index and Shannon index can reflect the level of biodiversity in a region. The existence of a larger value points out a higher community diversity. The Chao1 index is also used to indicate the number of OUT in a sample. A larger value of the Chao1 index indicates a greater number of species trees in the sample. This work showed that there is no significant difference in the bacterial diversity index under different nitrogen application levels. As the amount of nitrogen application increased, a trend of first increasing and then decreasing was recorded. It is also worth paying attention to the diversity of the soil bacteria under level II and level IV nitrogen application levels. The diversity of the soil bacteria under the level III nitrogen fertilization level was higher than that of the control. The type of nitrogen fertilizer application also has a certain impact on bacterial diversity. Previous meta-analysis works have also found that the application of ammonium nitrate did not significantly change the bacterial diversity of forest soil (Wang et al., 2023b). Considering that ammonium nitrate is easier for plants to absorb and utilize, it will not cause nitrogen fertilizer to be retained in the soil thus inducing soil acidification. In addition, the nitrogen application time in this work was short, so it failed to have a significant impact on bacterial diversity. In our work, a high nitrogen input had a tendency to reduce bacterial diversity. In general, long-term nitrogen addition will reduce bacterial diversity. After nitrogen addition, microorganisms will be subject to aggravated environmental stress. Most bacteria have a negative impact on soil pH. The adaptive range is poor, which will lead to a reduction in bacterial diversity (Wang et al., 2018b).

The bacterial α diversity did not significantly differ between the different nitrogen fertilization levels. Nonetheless, the composition of dominant soil bacteria changed between the different nitrogen fertilization treatments in our work. These results are consistent with the reported outcomes of He (He et al., 2023), reporting that no significant changes in microbial community diversity, composition, or structure were observed after 6 years of N addition. The duration of the N application may be one possible reason for this effect (Zheng et al., 2022). The nitrogen application time was also too short in our work. A previously reported work in the literature determined that the negative effects of N deposition on the microbial community were enhanced with the duration of experimental N input (Wang et al., 2018a). *Acidobacteria*, *Proteobacteria*, and *Chloroflexi* were the main dominant phyla. Previously, it was found that these bacteria were common dominant bacteria when nitrogen fertilizer was applied to acid soil in subtropical areas (Nie et al., 2018; Wang et al., 2018d,^2021^; Xi et al., 2022). Numerous works have demonstrated that adding nitrogen fertilizer could increase the abundance of bacteria with a nutrient rich lifestyle and decrease the abundance of bacteria with a nutrient poor lifestyle (Nie et al., 2018; Wang et al., 2018d,^2021^). However, our work found that with the increase in nitrogen content, the abundance of some oligotrophic bacteria (*Acidobacteria*) yielded an upward trend, while the abundance of eutrophic bacteria (*Actinobacteria* and *Proteobacteria*) showed a downward trend. This result is consistent with the results of Xi (Xi et al., 2022), where nitrogen addition experiments were conducted. The authors found some bacterial phyla are more sensitive to the influence of soil physical and chemical properties than their own trophic types in subtropical areas. Nitrogen addition experiments in temperate regions also found that *Acidobacteria* and *Chloroflexi* increased after nitrogen addition, while *Actinobacteria* decreased with nitrogen addition (Weng et al., 2023). *Acidobacteria* is a kind of acidophilic bacteria (Weng et al., 2023), which is usually negatively correlated with pH (Figure 6). The addition of nitrogen, especially high nitrogen, significantly reduced soil pH, resulting in an increase in the abundance of *Acidobacteria*. The best growth range of soil bacteria needs to be within a certain range of pH. After nitrogen application, the content of pH decreased, and the living conditions of soil bacteria were more severe, which could interpret the decrease in the abundance of *Proteobacteria* (Wang et al., 2018c,2023a).

### 4.3 Relationship between the soil physical and chemical properties and bacterial community

Soil bacterial community structure is closely related to the soil physical and chemical properties (Wang et al., 2018c; Yang et al., 2020, 2022). According to the literature, the soil total nitrogen content, pH and electrical conductivity are important factors leading to changes in soil bacterial communities (Ma et al., 2023). Our work has proven that soil environmental factors can significantly affect bacterial communities. Soil total nitrogen and pH are key factors that drive bacterial communities. Total nitrogen has a significant positive correlation with *Acidobacteria*, *Elusimicrobia*, and *Verrucomicrobia*, and pH has a significant positive correlation with *Chloroflexi*, *Firmicutes*, and *Bacteroidetes*. The total nitrogen content of soil had multiple effects on microbial growth, composition, and function. The soil pH could affect the microbial structure by changing the enzyme activity and controlling the suitable range for microbial growth (Wang et al., 2018b; Ma et al., 2023).

## 5 Conclusion

Nitrogen addition had a significant impact on some chemical properties. Under the level IV (150 gN⋅m^–2^⋅a^–1^) of nitrogen application level, the soil organic carbon and total nitrogen were significantly higher than the control, while soil pH, DOC, and total phosphorus were significantly lower than the control. The bacterial community was mainly composed of *Acidobacteria*, *Proteobacteria*, and *Chloroflexi*. With the increase of the nitrogen application level, the abundance of *Acidobacteria* increased and the abundance of *Proteobacteria* decreased. pH is significantly negatively correlated with the soil total nitrogen and nitrate nitrogen. Moreover, the soil organic carbon and organic matter are significantly positively correlated with nitrate nitrogen, and no significant correlation between the soil chemical properties and bacterial diversity was detected. The soil total nitrogen and pH were found to be the key driving factors for bacterial communities. Nitrogen addition affected the soil bacterial communities by regulating soil nitrogen and pH. The short-term nitrogen addition under the forest was studied in this work, while canopy nitrogen addition can better reflect the actual nitrogen deposition situation. Long term nitrogen application needs to be tracked and monitored, and a canopy nitrogen addition experimental platform should be established in the central and subtropical regions.

## Data availability statement

The datasets presented in this study can be found in online repositories. The names of the repository/repositories and accession number(s) can be found in this article/supplementary material.

## Author contributions

XJ: Writing – original draft, Visualization, Formal analysis, Software, Data curation. ZO: Data curation, Formal analysis, Writing – review & editing. CT: Data curation, Resources, Writing – review & editing. QH: Methodology, Project administration, Writing – review & editing. WZ: Conceptualization, Data curation, Formal analysis, Funding acquisition, Investigation, Methodology, Project administration, Resources, Software, Supervision, Validation, Visualization, Writing – original draft, Writing – review & editing. YT: Formal analysis, Methodology, Project administration, Writing – original draft. FH: Methodology, Writing – original draft. HS: Data curation, Writing – original draft.
